# Truly Universal Dental Cements vs. Conventional Ones—Comparison of Mechanical Properties

**DOI:** 10.3390/ma19102050

**Published:** 2026-05-14

**Authors:** Patrycja Starzyńska, Agata Szczesio-Wlodarczyk, Kinga Bociong

**Affiliations:** 1 Department of Oral Mucosal and Periodontal Diseases, Medical University of Lodz, 92-213 Lodz, Poland; 2 University Laboratory of Materials Research, Medical University of Lodz, 92-213 Lodz, Poland; 3 Department of General Dentistry, Medical University of Lodz, 92-213 Lodz, Poland

**Keywords:** cement, resin-based, universal, flexural strength, diametral tensile strength, hardness

## Abstract

This article evaluates the mechanical performance of truly universal resin cements, which promise broad substrate compatibility, simplified self-adhesive dual-cure mechanisms, and functional monomers. It addresses the inconsistent classification of market materials labelled as ‘universal’ and the lack of clear guidelines. Flexural strength (FS), diametral tensile strength (DTS), and Vickers hardness (HV) of ten resin cements were evaluated: five classified as truly universal (RelyX Universal, Calibra Universal+, Nexus Universal, Panavia SA, SoloCem) and five conventional (Panavia V5, RelyX U200, Cem SE DC, G-Cem One, Maxcem Elite). Significant variations occurred across the tested materials. RelyX Universal exhibited the highest HV (68), while Panavia V5 the lowest (20). FS ranged from 73.0 MPa (Panavia SA) to 114.4 MPa (G-Cem One). Elastic modulus values were lowest for RelyX Universal (3638 MPa) and Panavia SA Cement Universal (3812 MPa), and highest for Cem SE DC (8092 MPa). DTS values were comparable across the groups (35.4–50.2 MPa). No clear differences were observed between cements classified as truly universal and conventional materials in terms of FS, DTS, or HV. However, significant variations were found among individual materials (*p* < 0.05), indicating that mechanical performance is material-dependent rather than classification-based. Greater transparency in product characterisation is essential to enable accurate material classification.

## 1. Introduction

In recent years, dental adhesive technologies have evolved significantly, giving rise to materials that simplify procedures while enhancing clinical outcomes [1]. Among these advancements, universal resin cements have gained widespread acceptance as a reliable solution for a variety of indirect restorative applications [2]. The term “universal” reflects the versatility of these cements, which are designed to be compatible with a range of substrates—including ceramics, metal, and composite materials—and to bond effectively without the need for separate primers or bonding agents in many cases [3]. Universal cements aim to streamline workflows and reduce the complexities traditionally associated with dental adhesive systems, thereby improving efficiency and predictability in clinical practice [4].

Today, cements have been engineered with specific formulations to meet the demands of both practitioners and patients [5]. These products shall combine a balance of strength, ease of handling, and aesthetic quality that supports their use in many different types of restorations, from crowns and bridges to inlays, onlays, and veneers [6]. Each of these cements is designed to address common clinical challenges by offering durable adhesion, stable aesthetics, and user-friendly application [7].

However, despite their often shared “universal” designation, these cements differ in terms of composition, adhesive mechanisms, curing methods, and performance characteristics [8]. It means there are still no sufficient guidelines for defining the term “universal”.

Some articles suggest that truly universal cements should possess the following characteristics [9]:

- Versatility in protocols: Suitable for both self-adhesive and adhesive luting techniques, allowing adaptation based on clinical conditions and clinician preference.
- Broad substrate compatibility: Applicable for luting to both tooth tissues and all types of restorative materials.
- Integrated adhesive system: The cement system should include a universal adhesive that can function as a primer for tooth structures and/or restorative materials, eliminating the need for additional priming agents.
- Chemical bonding capability: At least one component of the cement/adhesive system should include functional acidic monomers and, ideally, silane-coupling agents to facilitate chemical bonding with both tooth tissues and restorative surfaces.
- Dual-curing properties: The material should be dual-cure (both light- and self-curing) to ensure optimal polymerisation and clinical performance, even in areas with limited light access.

Yet there are still too few references describing the problem of truly universal cements to base a thesis on it.

Truly universal materials are defined as resin cements that combine dual-cure capability, which allows them to be light-cured or self-cured depending on the clinical situation [10]. This flexibility is particularly valuable in scenarios where light penetration is limited, such as in posterior regions or under opaque restorations [11]. Additionally, many universal cements are formulated with functional monomers, such as 10-methacryloyloxydecyl dihydrogen phosphate (10-MDP), which chemically bond to tooth structure and restorative materials, providing long-term durability and stability [12]. These formulations are typically designed to be moisture-tolerant, reducing the risk of failure due to technique sensitivity, a common issue in adhesive dentistry [13].

Given these products’ unique properties, it is essential to evaluate their composition, bond strength, handling characteristics, and clinical performance to understand how each may be best applied in specific restorative scenarios [14].

This article aims to provide an in-depth comparison of these five universal cements by examining their flexural strength (FS), diametral tensile strength (DTS), and hardness (HV). By exploring above pointed factors, we aim to assist clinicians in selecting the optimal cement for a given application, ultimately contributing to successful, long-lasting restorations [15]. As well, the research aims to standardise the term “universal”. The posed null hypothesis of the study—there is no significant difference between truly universal cements and other cements available on the market.

## 2. Materials and Methods

The samples of truly universal cements and other selected cements were prepared in silicone moulds and then polymerised according to the manufacturer’s recommendations (Table 1). The cuing procedure [The Cure—TC—01, Spring Health Products (Norristown, PA, USA), 1400 mW/cm^2^ intensity] was performed after 2 min of self-polymerisation. The prepared materials were placed in distilled water at 37 °C for 24 h before testing. The Vickers hardness (HV), flexural strength (FS), and diametral tensile strength (DTS) were evaluated as indicators of resistance to functional loading, crack propagation, and surface durability in luting applications.

### 2.1. Hardness

Hardness of the tested resin cements was evaluated using the Vickers method with a Zwick ZHVµm hardness tester (Zwick–Roell, Ulm, Germany). A 1000 g load was applied for 10 s. Nine indentations were made on three randomly chosen specimens from the nine DTS samples in each study group.

Using nine Vickers indentations per group is consistent with previously published protocols for experimental dental composites and provides sufficient repeatability and precision of hardness values for this type of in vitro testing [16,17,18].

### 2.2. Flexural Strength

Rectangular specimens with dimensions of 2 mm × 2 mm × 25 mm were prepared for flexural strength (FS) testing, with six samples per group (n = 6 per group). The flexural strength was evaluated using a three-point bending test performed on a Zwick Roell Z020 universal testing machine (Zwick–Roell, Ulm, Germany) at a crosshead speed of 1 mm/min.

FS tests were carried out on the basis of the ISO 4049 standard [19], according to its assumptions, five samples are needed for testing; however, to further increase data reliability, we decided to make one additional.

### 2.3. Diametral Tensile Strength

Cylindrical specimens with a diameter of 6 mm and a height of 3 mm were tested for diametral tensile strength (n = 9 per group) using a Zwick Roell Z020 universal testing machine (Zwick–Roell, Ulm, Germany) at a cross-headspeed of 2 mm/min.

The use of nine cylindrical specimens per group is in line with another works focusing on mechanical characterisation of resin-based dental materials and is sufficient to obtain representative mean DTS values and ensure repeatable results [16,19,20,21].

### 2.4. Statistical Analysis

Statistical analyses were carried out using Statistica 13.1 software (Statsoft, Kraków, Poland). Data distribution normality was assessed with the Shapiro–Wilk test, whereas homogeneity of variances was examined using Levene’s test. For data approximating a normal distribution with equal variance homogeneity, parametric tests were employed, specifically one-way analysis of variance (ANOVA) followed by Fisher’s least significant difference post hoc tests. Data that did not meet these assumptions were analysed using non-parametric methods, namely the Kruskal–Wallis test with multiple comparisons of mean ranks. A predetermined significance level of *p* = 0.05 was applied.

## 3. Results

Descriptive statistics of experimental data can be found in Supplementary Files (Table S1). 

### 3.1. Hardness

The highest hardness values were observed for the RelyX Universal material, with a mean hardness of 68 HV. In contrast, the lowest mean values (20 HV) were recorded for Panavia V5 cement. Statistical analysis revealed the presence of significant differences between groups, as follows:

- Panavia V5 vs. Calibra Universal (*p* = 0.014081), Nexus Universal (*p* = 0.005459), Cem SE DC (*p* = 0.000000), G-Cem One (*p* = 0.000042), Maxcem Elite (*p* = 0.027318), RelyX U200 Automix (*p* = 0.000000),
- Panavia SA Cement Universal vs. Cem SE DC (*p* = 0.000220), G-Cem One (*p* = 0.011619), RelyX U200 Automix (*p* = 0.00005),
- RelyX Universal vs. Cem SE DC (*p* = 0.000032), G-Cem One (*p* = 002385), RelyX U200 Automix (*p* = 0.000001),
- SoloCem vs. Cem SE DC (*p* = 0.023903), RelyX U200 Automix (*p* = 0.001226).

While RelyX Universal exhibited the highest hardness (68 HV), all tested materials fell within a range common for resin-based luting agents, suggesting that, despite statistical variation, these materials may offer acceptable surface resistance for various indirect restoration types.

**Figure 1:**
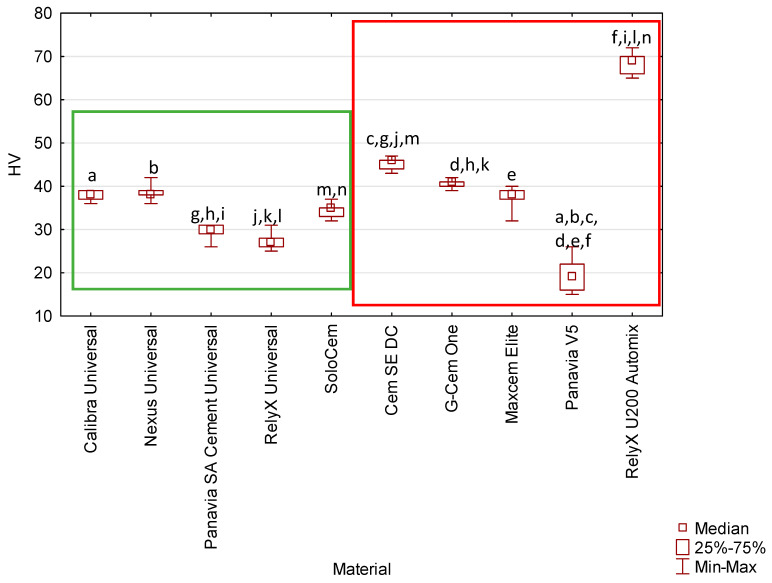
Median values of Vickers hardness of tested cements. Identical superscript letters demonstrate statistically significant differences (*p* ≤ 0.05). Cements classified as truly universal are enclosed within the green box, while other tested cements are framed in red.

The median hardness values for all tested materials are shown in Figure 1.

### 3.2. Flexural Strength

**Figure 2:**
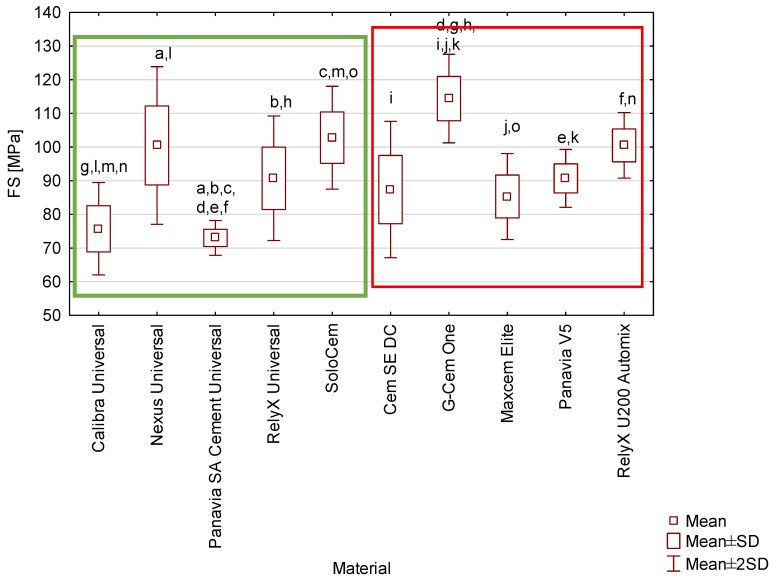
Mean values of flexural strength of tested cements. Identical superscript letters demonstrate statistically significant differences (*p* ≤ 0.05). Cements classified as truly universal are enclosed within the green box, while other tested cements are framed in red.

**Figure A1:**
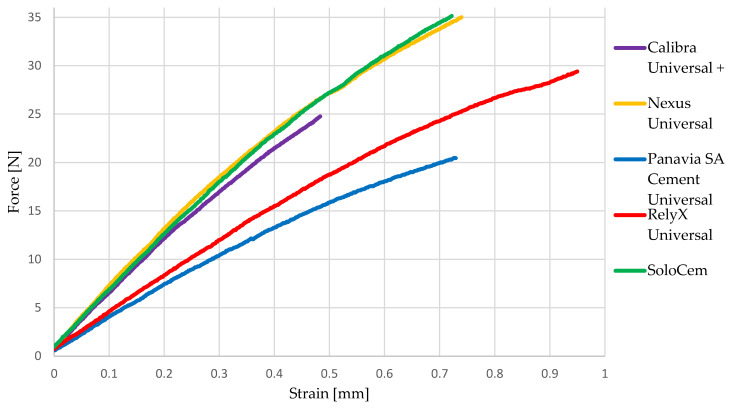
Representative force–strain curves from flexural strength testing for the five cements classified as truly universal.

**Figure A2:**
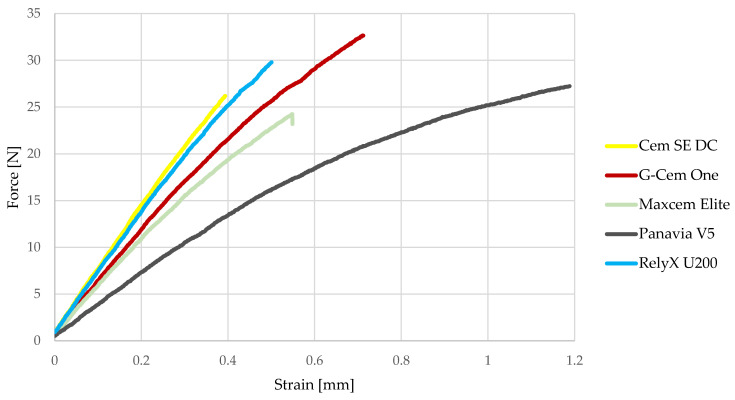
Representative force–strain curves from flexural strength testing for the five other tested cements.

The recorded mean three-point flexural strength values ranged from 73.0 MPa for Panavia SA Cement Universal to 114.4 MPa for G-Cem One (Figure 2). The force–strain curves from flexural strength testing for the cements are in the Appendix A (Figure A1 and Figure A2). Post hoc comparisons demonstrated statistically significant differences among the following groups (*p* < 0.05):

- Panavia SA Cement Universal vs. Nexus Universal (*p* = 0.000187), RelyX Universal (*p* = 0.018640), SoloCem (*p* = 0.000163), G-Cem One (*p* = 0.000158), Panavia V5 (*p* = 0.019063), RelyX U200 Automix (*p* = 0.000187),
- G-Cem One vs. Calibra Universal (*p* = 0.000158), RelyX Universal (*p* = 0.000604), Cem SE DC (*p* = 0.000198), Maxcem Elite (*p* = 0.000166), Panavia V5 (*p* = 0.000592),
- Calibra Universal vs. Nexus Universal (*p* = 0.000372), SoloCem (*p* = 0.000198), RelyX U200 Automix (*p* = 0.000369),
- SoloCem vs. Maxcem Elite (*p* = 0.021555).

While G-Cem One exhibited the highest mean flexural strength (114.4 MPa), all materials evaluated in this study remained within the range typically associated with resin-based luting systems. The observed statistical variations highlight that individual formulation differences exert a more significant influence on flexural resistance than the ‘universal’ classification.

**Figure 3:**
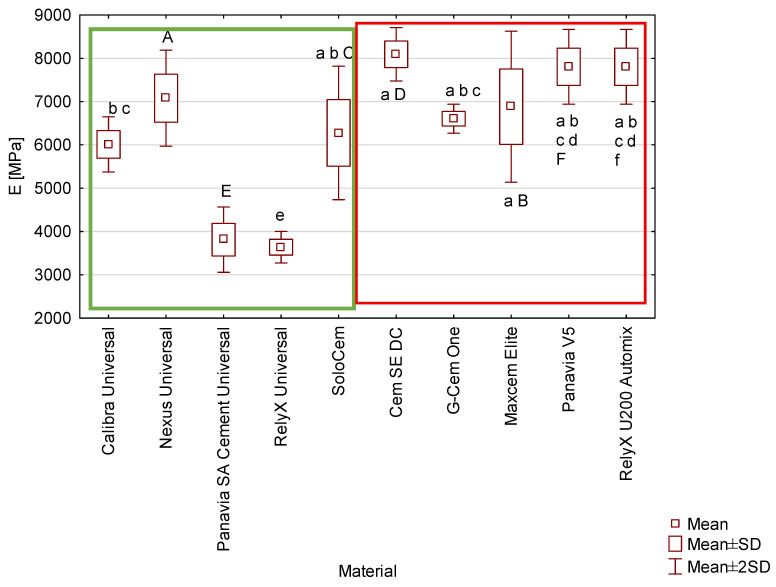
Mean values of flexural modulus of tested cements. There are no statistically significant differences (*p* > 0.05) between the material marked with a capital letter and the materials marked with the same lowercase letter. Cements classified as truly universal are enclosed within the green box, while other tested cements are framed in red.

The lowest elastic modulus values were observed for RelyX Universal (3638 MPa) and Panavia SA Cement Universal (3812 MPa), whereas the highest values were recorded for Cem SE DC (8092 MPa) (Figure 3). Most differences were statistically significant (*p* ≤ 0.05). *No significant* differences were found between the following materials (*p* > 0.05):

- Nexus Universal vs. SoloCem, Cem SE DC, G-Cem One, Maxcem Elite, Panavia V5, RelyX U200 Automix,
- Maxcem Elite vs. Calibra Universal, SoloCem, G-Cem One, Panavia V5, RelyX U200 Automix,
- SoloCem vs. Calibra Universal, G-Cem One, Maxcem Elite,
- Cem SE DC vs. Panavia V5, RelyX U200 Automix,
- Panavia SA Cement Universal vs. RelyX Universal,
- Panavia V5 vs. RelyX U200 Automix.

### 3.3. Diametral Tensile Strength

**Figure 4:**
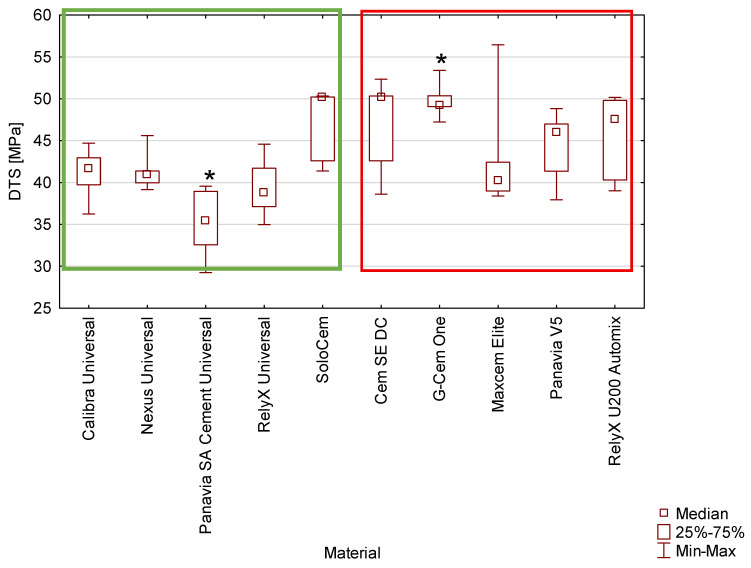
Median values of diametral tensile strength of tested cements. *—indicate statistically significant differences (*p* ≤ 0.05). Cements classified as truly universal are enclosed within the green box, while other tested cements are framed in red.

**Figure A3:**
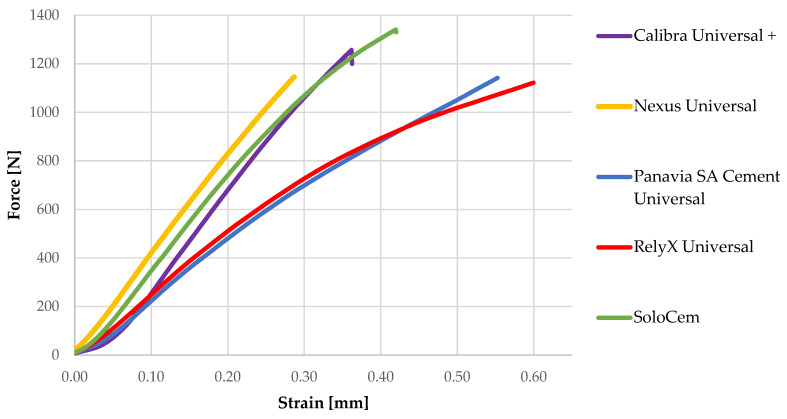
Representative force–strain curves from diametral tensile strength testing for the five cements classified as truly universal.

**Figure A4:**
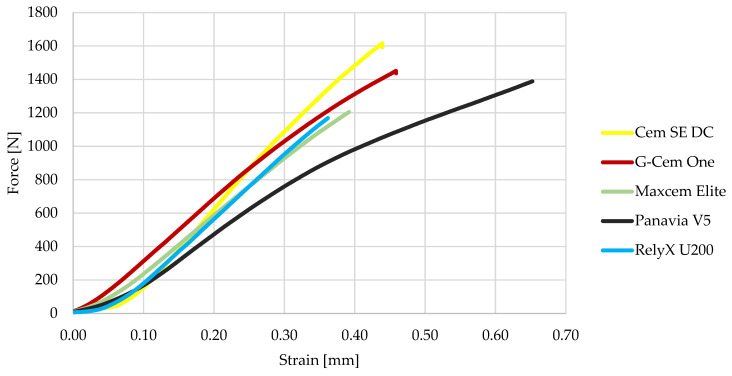
Representative force–strain curves from diametral tensile strength testing for the five other tested cements.

The force–strain curves from diametral tensile strength testing for the cements are in the Appendix A (Figure A3 and Figure A4). The analysis demonstrated that, for most of the tested cements, the DTS values did not differ significantly (Figure 4). However, G-Cem One showed a significantly higher DTS value compared to Panavia SA Cement Universal (*p* < 0.05). The recorded DTS values ranged from 35.4 MPa to 50.2 MPa across the investigated materials.

Notably, the results indicate a generally comparable tensile performance among the majority of the cements.

## 4. Discussion

The diverse formulations of universal cements contribute to distinct profiles of clinical performance, each presenting specific advantages and potential limitations [22]. Some cements are engineered to emphasise procedural simplicity, addressing the preferences of practitioners seeking reliable [23] and uncomplicated application techniques. Others are developed with an all-in-one strategy, achieving high bond strengths without necessitating an additional adhesive step in most instances. Particular materials exhibit enhanced bonding capabilities to ceramic substrates, while others are valued for their ease of manipulation and established track record in restorative dentistry [24]. In addition, certain resin cements are designed to accommodate an extensive range of indirect luting procedures, with some incorporating universal, dual-curing, self-adhesive formulations that aim to streamline clinical protocols. Unlike conventional multi-step resin cements (e.g., Panavia V5), truly universal formulations like RelyX Universal and Panavia SA Universal integrate silane and 10-MDP for primer-free adhesion, though in vitro studies show no consistent superiority in bond strength over primed systems. In addition, certain resin cements are designed to accommodate an extensive range of indirect luting procedures, with some incorporating universal, dual-curing, self-adhesive formulations that aim to streamline clinical protocols [25].

The evolution of universal resin cements has offered clinicians a versatile range of options for indirect restorative procedures. Designed to simplify the clinical workflow by eliminating the need for separate primers or bonding agents, these materials are marketed as compatible with a broad spectrum of restorative substrates. However, the term “universal” remains inadequately defined in both scientific literature and commercial usage as highlighted by Maravić et al., who propose classifying them as distinct from self-adhesive cements due to optional adhesive modes. Truly universal resin cements are characterised by: self-etching or universal adhesive functionality via acidic monomers (e.g., 10-MDP, carboxylic acid monomers), compatibility with diverse substrates (enamel, dentin, ceramics, metals, composites) without preconditioning, silanisation, or metal primers and dual-cure activation for reliable polymerisation [26]. Following the approach suggested by Maravić et al., the study compared five materials considered truly universal resin cements (RelyX Universal, Calibra Universal+, Nexus Universal, Panavia SA Cement Universal, SoloCem) with five other resin cements that do not meet these criteria (Panavia V5, RelyX U200 Automix, Cem SE DC, G-Cem One, Maxcem Elite), as the latter require dedicated primers (e.g., Panavia V5 tooth primer), exhibit substrate-dependent bond strengths (e.g., lower on dentin or specific ceramics), or show predominant adhesive failures at the cement–dentin interface [3]. Mechanical testing did not reveal a clear separation between the “truly universal” group and the other materials tested. The observed differences are most likely related to the specific formulation and composition of each material.

The specific clinical implications of the substantial variation in mechanical properties remain unclear. In well-executed tooth preparations with adequate resistance and retention form, these differences may have minimal impact. However, in preparations lacking sufficient resistance and retention, or in nonretentive designs such as tabletop ceramic onlays, the effects could be clinically significant [27]. An ideal dental cement must possess adequate mechanical properties to withstand functional forces throughout the lifespan of the restoration. It should also resist degradation in the oral environment and maintain strong adhesion to the underlying dentin [28]. Long-term clinical success depends on the cement’s ability to withstand fracture and cyclic fatigue. A wide range of mechanical properties—including compressive strength, flexural strength, diametral tensile strength, elastic modulus, fracture toughness, hardness, creep behaviour, and thermal effects—have been examined to better understand cement behaviour [29]. However, studies directly comparing the new universal cements are still lacking.

The mechanical properties of the various resin-based cements tested differed (Figure 1, Figure 2, Figure 3 and Figure 4). However, the underlying factors responsible for these variations have not been fully elucidated. It is well established that the behaviour of composite materials is closely linked to their chemical composition [30]. These materials are complex mixtures containing chemical primers, viscosity modifiers, filler particles, and silanes. For the tested commercially available resin cements, assessing this relationship is hindered by manufacturers’ limited compositional data. The lack of detailed, transparent compositional data substantially limits the ability to assess the influence of individual constituents on the mechanical and clinical performance of these materials. Accordingly, there is a clear need for comprehensive fundamental research employing experimental cements with fully defined compositions and dual-cure polymerisation systems. Such investigations would provide a more reliable basis for identifying which specific components—and to what extent—they contribute to the observed differences in mechanical properties among materials marketed as “truly universal,” as compared with other resin-based cements used in contemporary dental practice.

Flexural strength, as evaluated in this study, refers to a material’s ability to withstand deformation under load, incorporating both compressive and tensile stresses. Flexural modulus describes the stiffness of a material when it is bent [31]. Sufficient flexural strength and modulus allow stresses to be transferred or moderated between the restoration and the tooth structure without causing fracture or permanent deformation, thereby increasing the restoration’s resistance to failure and protecting brittle restorative materials. Flexural strength of dental cements is typically evaluated using a three-point bending test in accordance with ISO 4049. From a materials science perspective, these cements can also be classified as composite materials, as they consist of a resin matrix reinforced with inorganic fillers [32]. Diametral tensile strength offers an indirect measure of tensile strength. For this test, the material is shaped into a disk and loaded along its diameter with compressive forces until fracture occurs [33].

Flexural strength (FS) and diametral tensile strength (DTS) results illustrate the mechanical disparities among the tested materials. The flexural strength values ranged from 73.0 MPa to 114.4 MPa, whereas the diametral tensile strength values varied between 35.4 MPa and 50.2 MPa. The values obtained in the present study fall within ranges that are comparable to those reported in other investigations [34,35].

G-CEM ONE exhibited the highest FS, E and DTS, suggesting excellent internal cohesion and elastic resistance. This effect may be due to the exceptionally high filler content. According to information provided to the authors [36] the material contains 70 wt. % of an inorganic phase. It has been demonstrated that, up to a certain threshold, flexural strength increases with higher filler loading in resin-based composite materials. It should be emphasised, however, that effective reinforcement requires a strong interfacial bond between the organic matrix and the inorganic fillers (e.g., silanisation), and the filler size and morphology are also very important aspects [37]. An additional innovation introduced by GC is the incorporation of a catalyst intended to generate a greater number of free radicals during the chemical polymerisation phase. In our investigation, the materials were allowed to self-cure for 2 min prior to light activation. According to the manufacturer’s internal data, in self-curing mode, this innovation results in higher flexural strength than other tested cements [38].

Elastic modulus and Vickers hardness values further illuminate the materials’ differences. High modulus values for the majority of tested cements indicate increased stiffness and resistance to deformation under stress. This characteristic is desirable in high-load-bearing restorations such as posterior crowns or fixed bridges [39]. However, it should be noted that not all the materials exhibit high strength and hardness. The relationship between mechanical properties (e.g., elastic modulus, hardness) and material composition is complex and multifactorial, especially for commercially available ones. Numerous factors—including the type and proportion of monomers, filler characteristics, and the photoinitiator system—contribute to material variability [40]. Panavia SA Universal and RelyX Universal had significantly lower modulus values, suggesting greater material flexibility. Although this could limit their utility under heavy occlusal forces, the elasticity may be advantageous in minimally invasive restorations where greater flexibility and improved stress distribution are essential.

Hardness is an important indicator of resistance to plastic deformation and is commonly used to evaluate the performance of resin-based materials. It is strongly influenced by filler loading, although factors such as filler morphology, coupling agent efficiency, and the degree of conversion also contribute to its final value [41,42,43,44]. Because hardness increases with monomer conversion, it is frequently used as an indirect measure of polymer network development. Furthermore, in resin composites, higher hardness has been associated with enhanced wear resistance [45]—a property that may also be relevant for resin cements [46]. In the present study, Panavia V5 showed the lowest hardness among the tested cements. Its relatively low filler content (38 wt. %) likely played a decisive role. Similar values were reported by Toida et al. [47], who additionally found that Panavia V5 exhibited higher bond strength, together with lower elastic modulus and hardness, compared with other cements. The authors suggested that a lower modulus may improve stress distribution under tensile loading, enhancing resistance to debonding; this may also be facilitated by the material’s monomer blend and lower viscosity, which allow better penetration and integration at the adhesive interface. Cohesive failure patterns further supported this interpretation. However, these findings must be considered in light of methodological differences in measuring modulus and hardness, and the fact that the cited study evaluated only three materials, underscoring the need for broader investigation.

PANAVIA SA Universal and PANAVIA V5 also performed well in mechanical testing, consistent with their 10-MDP content, which is known for enhancing chemical bonding to both tooth substrates and metal oxides [48,49]. In contrast, Maxcem Elite and Nexus Universal showed relatively lower diametral tensile and flexural strengths, which may reflect their simplified application protocols and reduced chemical interaction with substrates. While such materials may offer clinical convenience, these findings suggest a potential trade-off in mechanical robustness.

Our findings contribute to the ongoing effort to establish clear guidelines for the use of universal cements. Although the development of products designed to simplify cementation procedures is valuable, clinicians require evidence-based parameters for selecting the appropriate material. The significant variability in physical properties across commercially available cements underscores the need for standardised definitions and testing protocols. The inclusion of MDP monomers, silane agents, dual-curing efficiency, and validated bonding performance across multiple substrates should be considered essential criteria for a cement to be truly classified as “universal” [50,51].

Summarising while universal resin cements offer significant clinical advantages by reducing the complexity of restorative procedures, their performance characteristics differ markedly. This study reinforces the need for careful material selection based on specific clinical indications. It also calls for clearer nomenclature and performance standards within the industry. Future research should focus on developing comprehensive, standardised classifications for universal cements, ensuring both manufacturers and clinicians operate under shared definitions grounded in clinical efficacy and material science [52].

This in vitro study has limitations inherent to mechanical testing. First, short-term tests do not simulate long-term oral degradation (i.e., thermocycling, enzymatic hydrolysis), which reduces FS by 20–30% in resin cements. Second, ISO-compliant specimens do not account for substrate interactions and thin-film behaviour observed in vivo. Third, lack of fatigue-to-failure protocols limits extrapolation to clinical lifespans (>10^5^ cycles). Fourth, manufacturer opacity on compositions hinders causal analysis. Future work should include aged, bonded assemblies under dynamic loading [53].

## 5. Conclusions

In conclusion, the present in vitro study did not identify a clear separation in mechanical performance between the group of truly universal cements and the other tested materials. These results highlight the need for further fundamental research to clarify how specific additives, such as adhesive monomers and silanes, affect mechanical performance as a function of concentration. Moreover, greater transparency in product characterisation provided by manufacturers is essential to enable accurate material classification and to reduce the risk of errors in clinical practice.

## Figures and Tables

**Table 1:** Characteristics of the cements used in the study. *—composition and curing time obtained from Manufacturer’s information.

Material	Manufacturer	Composition *	Cure Time [s] *	Type
Calibra Universal +	Dentsply Sirona, Charlotte, NC, USA	PENTA, BIS-GMA, UDMA, TMPTMA, TEGDMA, Cumene hydroperoxide, acrylic acid, oxybenzone	20	Truly Universal Resin Cement
Nexus Universal	Kerr Corporation, Orange, CA, USA	Bisphenol A ethoxylate dimethacrylate, UDMA, TEGDMA, HEMA, SILANE A174, 1,1,3,3-tetramethylbutyl hydroperoxide	10	Truly Universal Resin Cement
Panavia SA Cement Universal	Kuraray Noritake Dental Inc. Okayama, Japan	BIS-GMA, TEGDMA, HEMA, sodium fluoride, silanated barium glass filler, silanated colloidal silica, 10-MDP, aluminium oxide filler, hydrophobic aromatic dimethacrylate Silane coupling agent, dl-Camphorquinone, Peroxide, accelerators, catalysts, pigments, filler load 62 wt. % (40 vol%)	10	Truly Universal Resin Cement
RelyX Universal	3M Deutschland GmbH Neuss—Germany	Glass powder, Silane A174, bulk material, substituted dimethacrylate, 1,12-dodecane dimethycrylate, 2,4,6(1H,3H,5H)-Pyrimidinetrione, 5-phenyl-1- (phenylmethyl)-, calcium salt (2:1), silane treated silica, calcium hydroxide, SPTS, NUC		Truly Universal Resin Cement
SoloCem	Coltène, Altstätten, Switzerland	TEGDMA, UDMA, BIS-GMA, HEMA, ZnO, Ytterbium fluoride, CAO 1, 10-MDP, G20, 4-meta	20	Truly Universal Resin Cement
Cem SE DC	ORBIS Dental Group San Pedro Avenue, San Antonio, TX, USA	Barium aluminium borosilicate glass, GlyDMA, UDMA, methacrylate phophoric acid ester, BisGMA, fumed silica, HPMA, BisEMA, initiators, stabilisers, pigments	20	Universal, Dual-Curing, Self-Adhesive, Luting Composite
G-Cem One	GC Corporation Tokyo, Japan	Dimethacrylate, UDMA, NUC, monomer, synergist, photoinitiator, stabiliser, initiator 70 wt. %, catalist of self-cure polymerisation more radicals	10	Universal Self-Adhesive Resin Cement
Maxcem Elite	Kerr Corporation, Orange, CA, USA	60% filler content, Fiber Glass Wool, Ytterbium fluoride, HDDMA, Light Ester G 101P, UDMA, SILANE A174, Fumed silica	10	Self-Etch, Self-Adhesive Dental Cement
Panavia V5	Kuraray Noritake Dental Inc. Okayama, Japan	BIS-GMA, TEGDMA, silanated barium glass filler, silanated fluoroalminosilicate glass filler, colloidal silica, surface treated aluminium oxide filler, hydrophobic aromatic dimethacrylate hydrophilic aliphatic dimethacrylate, dl-camphorquinone, initiators, accelerators, pigments filler load 61 wt. % (38 vol%)	10	Adhesive Resin Cement System
RelyX U200	3M Deutschland GmbH Neuss—Germany	Glass powder, SILANE A174, bulk material, substituted dimethacrylate, 1,12-dodecane dimethycrylate, 2,4,6(1H,3H,5H)-Pyrimidinetrione, 5-phenyl-1-(phenylmethyl)-, calcium salt (2:1), silane treated silica, calcium hydroxide, SPTS, NUC	20	Self-Adhesive Resin Cement

HDDMA—1,6 hexanediol dimethacrylate; UDMA—7,7,9(or 7,9,9)-trimethyl-4,13-dioxo-3,14-dioxa-5,12-diazahexadecane-1,16-diyl bismethacrylate; SILANE A174—3-trimethoxysilylpropyl methacrylate; Light Ester G 101P—2-hydroxy-1,3-propanediyl bismethacrylate; SPTS—sodium P-toluenesulfinate; NUC—titanium dioxide; BIS-GMA—bisphenol A diglycidylmethacrylate; TEGDMA—triethylene glycol dimethacrylate; HEMA—2-hydroxyethyl methacrylate; 10-MDP—10-methacryloyloxydecyl dihydrogen phosphate; ZnO—zinc oxide; CAO 1—2,6-di-tert-butyl-4-methylphenol; G20—dibenzoyl peroxide; 4-meta—4-methacryloxyethyl trimellitic anhydride; BIS-EMA—bisphenol A ethoxylate dimethacrylate; PENTA—dipentaerythritol pentaacrylate phosphate; TMPTMA—propylidynetrimethyl trimethacrylate; Cumene hydroperoxide—Į,Į-dimethylbenzyl hydroperoxide.

## Data Availability

The authors declare that the data supporting the findings of this study are available within the paper and its Supplementary Information files. Should any raw data files be needed in another format, they are available from the corresponding author upon reasonable request.

## Supplementary Materials

The following supporting information can be downloaded at: https://www.mdpi.com/article/10.3390/ma19102050/s1, Table S1: Raw experimental data (Excel file) ((a). flexural modulus; (b). diametral tensile strength; (c). Flexural Strength; (d). Vickers hardness).
